# Management of Methicillin-Resistant Staphylococcus aureus-infected femoral nonunion during lengthening in achondroplasia using circular external fixator: a case report

**DOI:** 10.1186/s12891-024-08224-0

**Published:** 2024-12-23

**Authors:** Koji Nozaka, Tsuyoshi Shirahata, Yusuke Yuasa, Naohisa Miyakoshi

**Affiliations:** Department of Orthopedic Surgery, Akita University Graduate School of Medicine, 1-1-1 Hondo, Akita, 010-8543 Japan

**Keywords:** Achondroplasia, MRSA, Infected femoral nonunion, Circular external fixator

## Abstract

**Background:**

Achondroplasia, the most common form of rhizomelic dwarfism, occurs in approximately 1 in 25,000 individuals. Clinical features include attenuated growth, rhizomelic limb shortening, and craniofacial abnormalities. Limb-lengthening surgery is widely employed to improve quality of life. However, reports on Methicillin-Resistant Staphylococcus aureus (MRSA) infections in femoral nonunions at lengthening sites are scarce.

**Case presentation:**

A 15-year-old boy with achondroplasia presented with MRSA-infected femoral nonunion. Bilateral femoral lengthening had been performed at age 13 using unilateral external fixators. Following a 7 cm lengthening of the right femur, surgical site infection occurred, with MRSA detected on postoperative day 127. Despite debridement and autologous iliac bone graft with non-locking screws, nonunion persisted. Referred to our hospital at age 15, the patient underwent radical debridement until punctate bleeding appeared, and vancomycin-loaded cement beads were implanted. A circular external fixator, effective even with bone weakened by prolonged non-weight bearing, was applied. Six weeks later, further debridement and vancomycin bead replacement were performed. Final fixation included refreshing the nonunion site and placing a cancellous bone graft from the contralateral iliac bone. Bone fusion progressed, and the ring was removed 9 months post-surgery. After seven years, no recurrence of infection was noted. Although slight knee flexion limitation persisted, the patient experiences no pain while walking and has become a healthy working adult.

**Conclusion:**

This case highlights the effectiveness of radical debridement, antibiotic-loaded cement beads, autologous bone grafting, and circular external fixation in treating MRSA-induced nonunion at femoral lengthening sites in achondroplasia. Circular external fixators provide stable fixation even in cases of prolonged bone weakness.

## Background

Achondroplasia is the most common form of rhizomelic dwarfism, with an incidence of approximately one in 25,000 people [1–5]. Clinical outcomes include attenuated growth, rhizomelic shortening of long bones, and craniofacial abnormalities. Currently, no pharmacological treatment exists for achondroplasia. However, surgical limb lengthening enhances patients’ well-being and daily function. Furthermore, bilateral lower limb lengthening is common and enhances the quality of life of selected patients with achondroplasia [2]. However, few reports exist, on infected femoral nonunions during lengthening in achondroplasia. Nonunion of femoral fractures in children is a rare but serious issue that is both devastating and disabling for patients, and challenging for orthopedic surgeons [6]. Treatment becomes more challenging if an associated infection occurs. Moreover, the situation becomes more complex with additional complications, such as disuse osteoporosis and scars from previous surgical interventions [3]. Due to the rarity of these fractures in children, no consensus exists on a standard treatment method for these cases.

## Case presentation

**Fig. 1:**
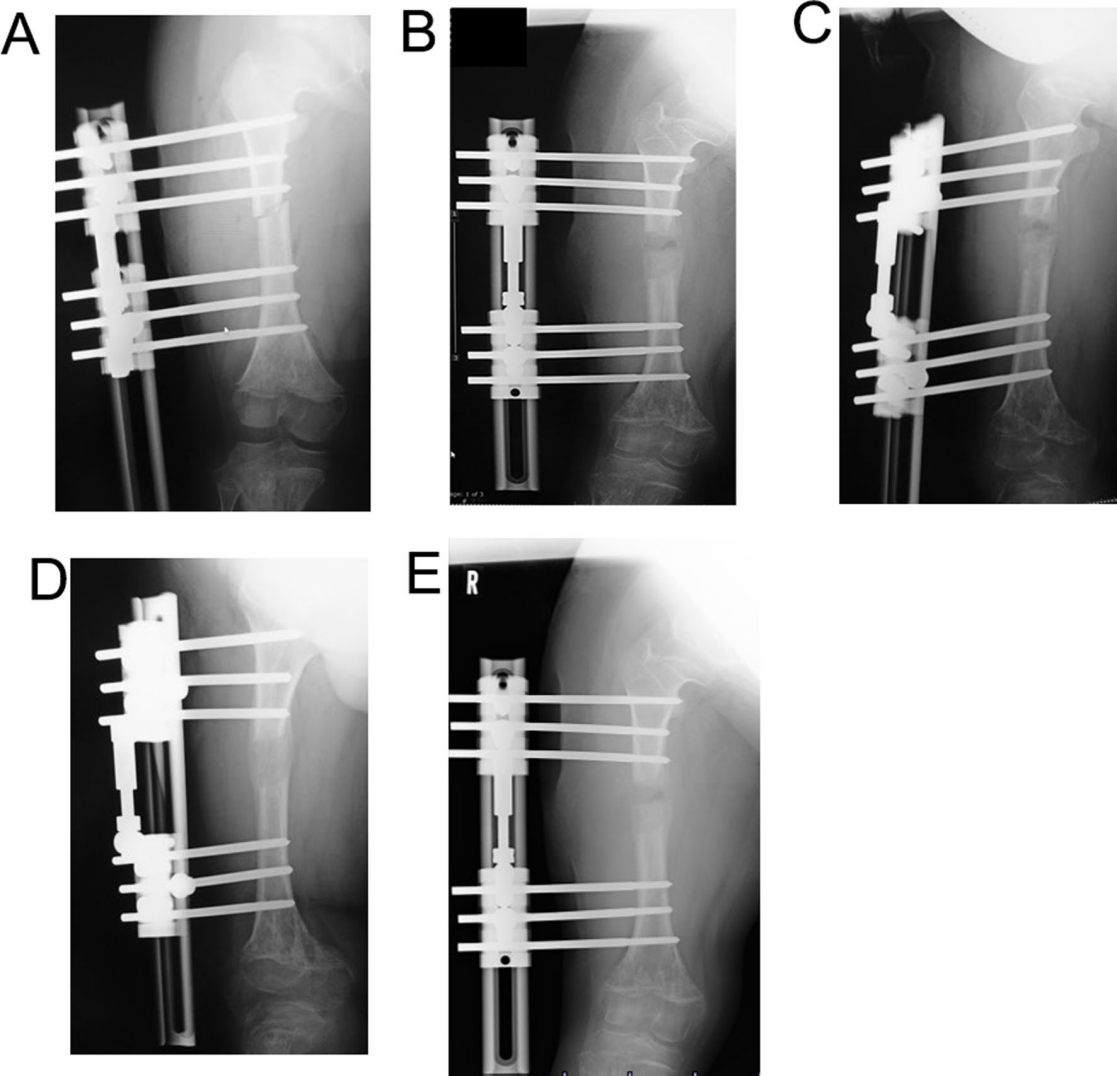
Postoperative X-ray of the femoral lengthening site that was treated using a unilateral external fixator in the previous hospital. **A**: Day 0. **B**: Day 30. **C**: Day 60. **D**: Day 120. **E**: Day 127

**Fig. 2:**
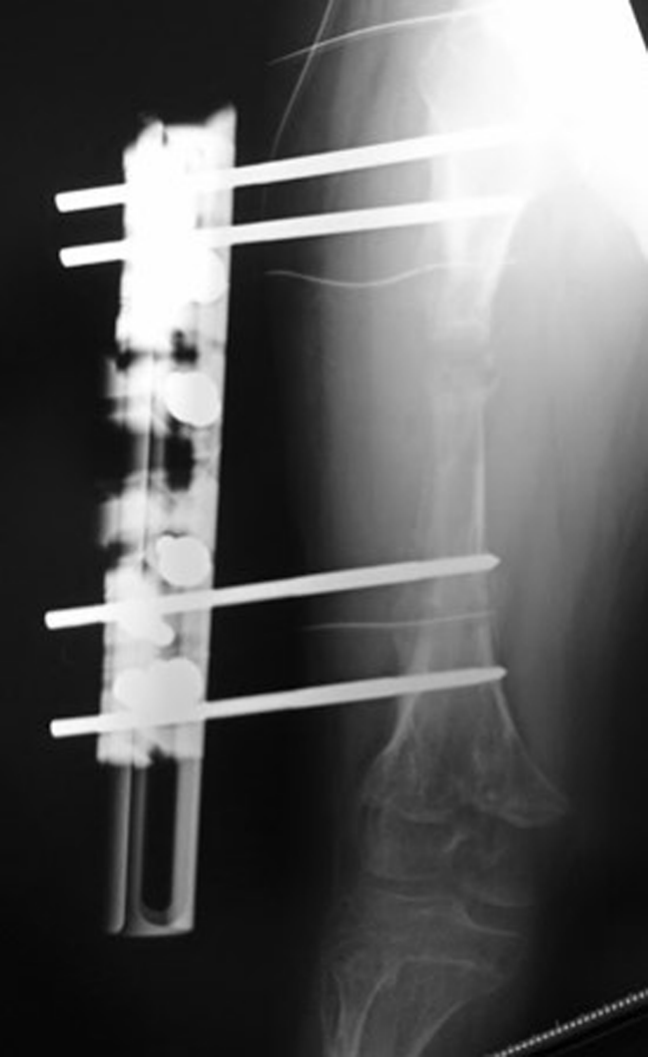
X-ray shows deep pin-tract infection 5 months after the initial surgery

**Fig. 3:**
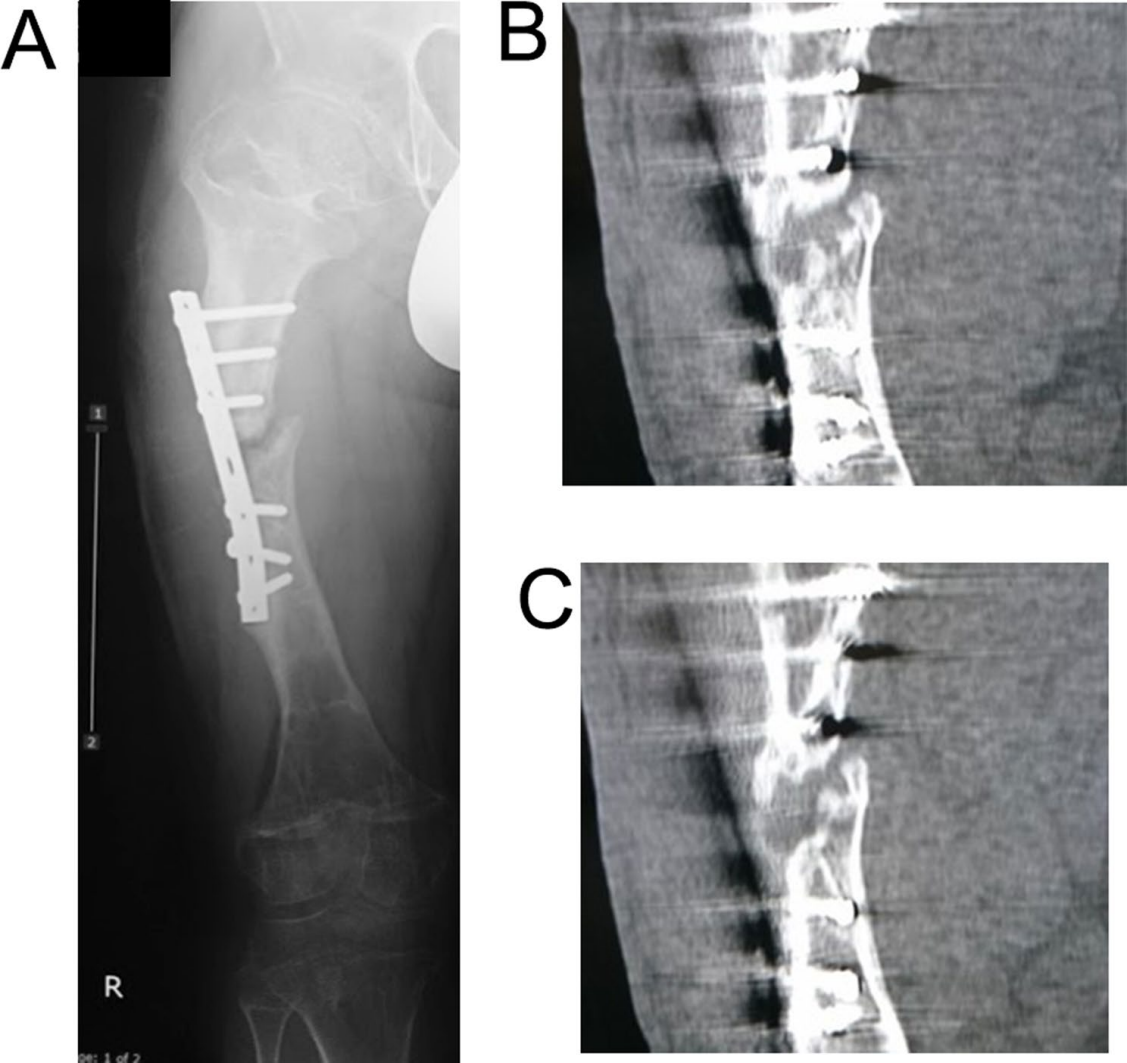
X-ray (**A**) and CT (**B**, **C**) show osteomyelitis and pathological fracture by deep pin-tract infection at the femur

**Fig. 4:**
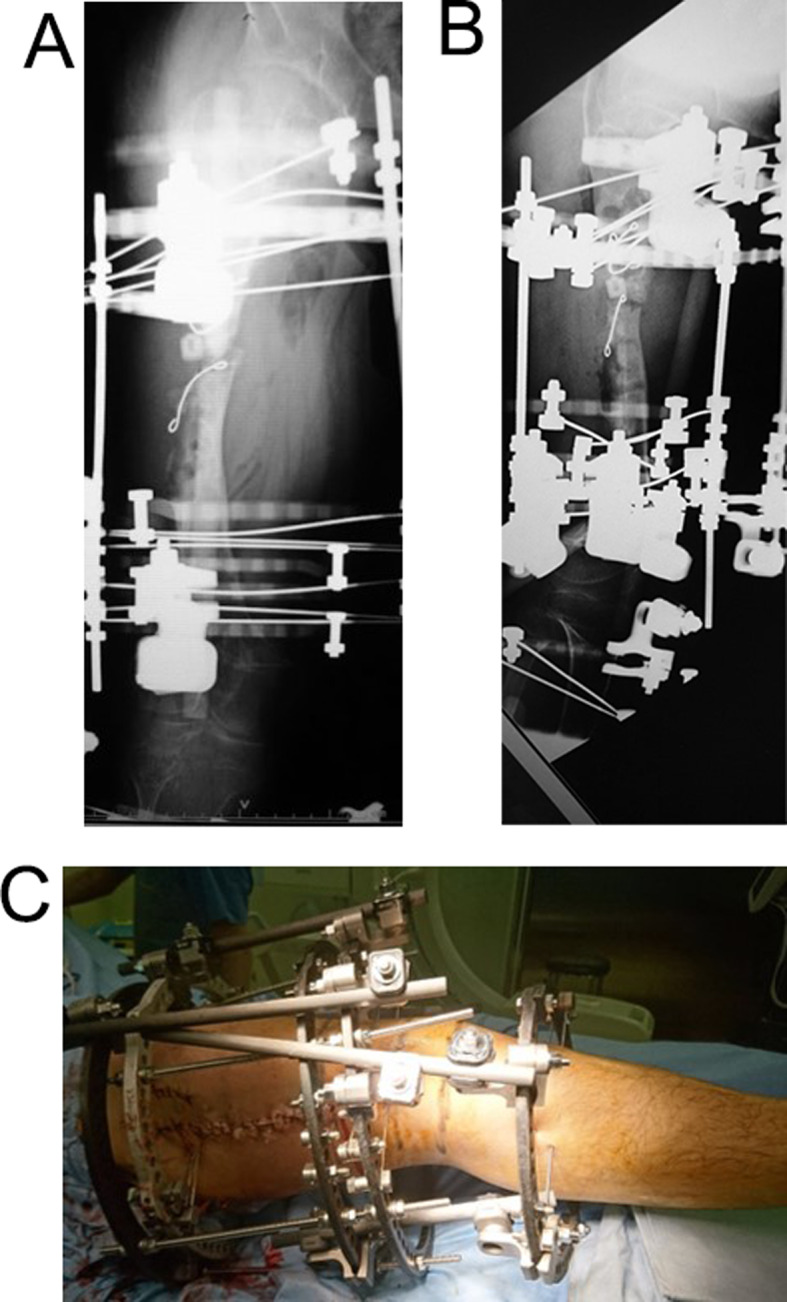
**A**, **B**: Immediate postoperative X-ray at initial surgery. **C**: A knee joint-spanning circular external fixator was used. The tibial ring was removed 2 weeks after surgery, and range-of-motion exercises were started

**Fig. 5:**
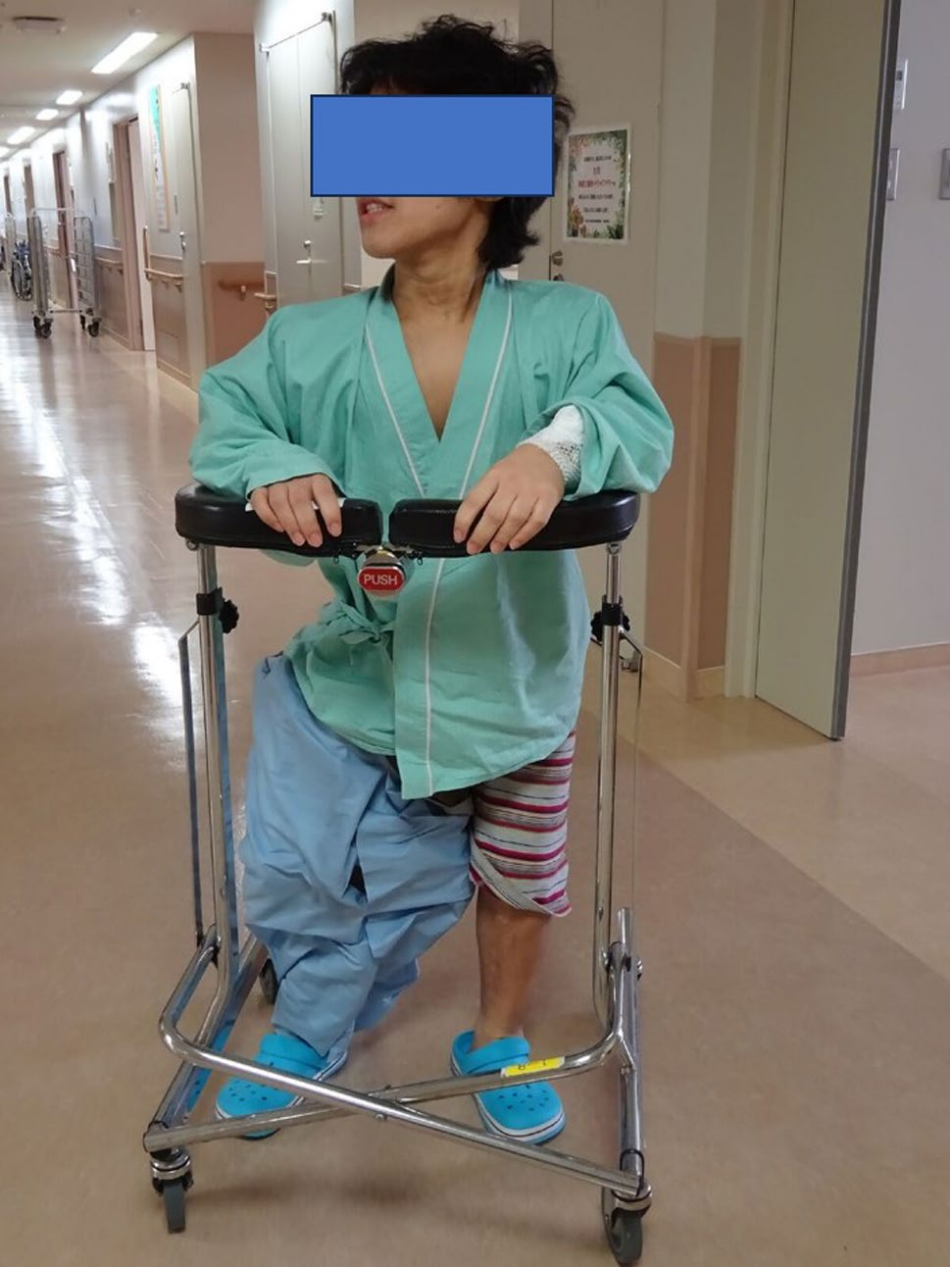
Partial weight bearing using a walker after surgery

**Fig. 6:**
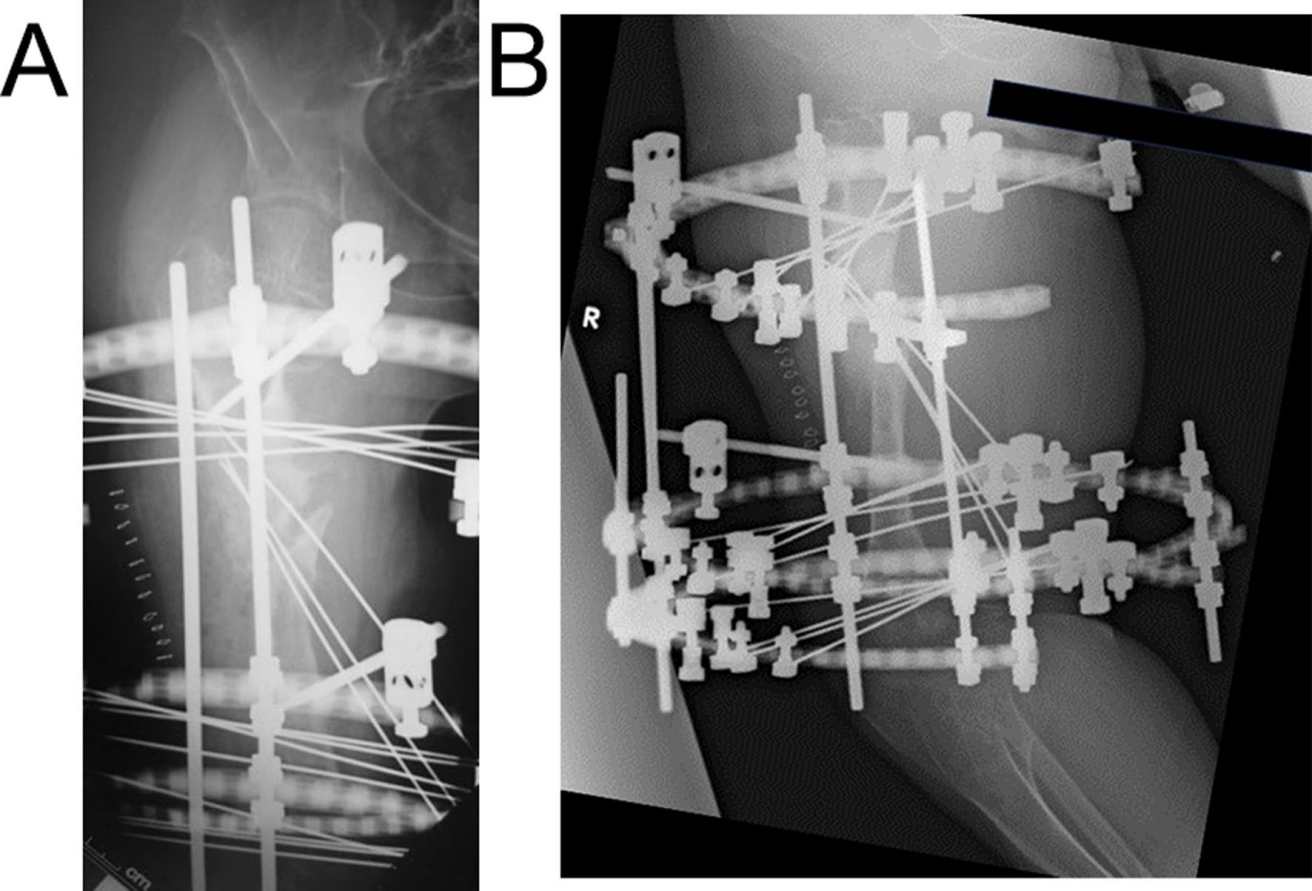
Third surgery postoperative X-rays

**Fig. 7:**
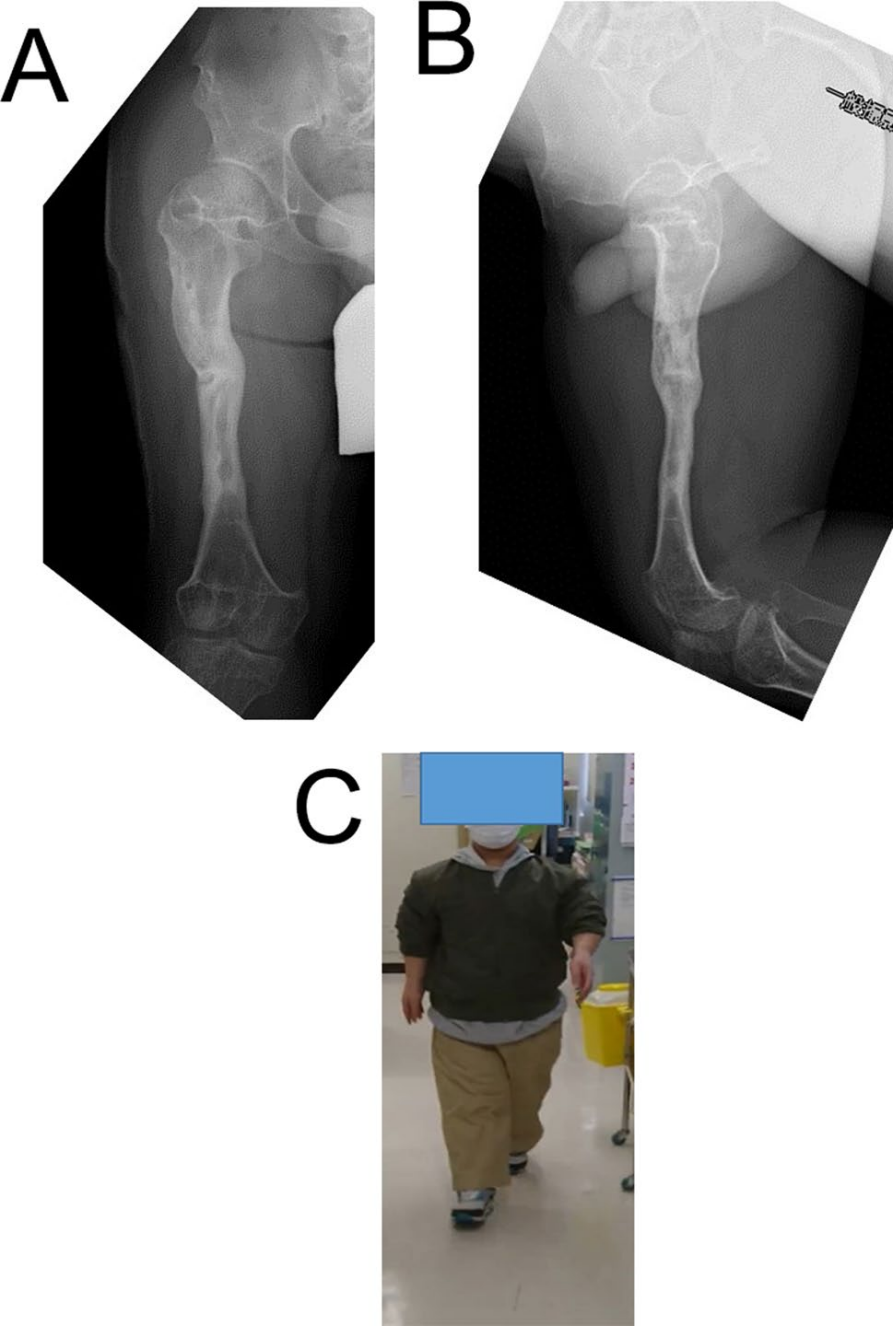
**A**, **B**: X-ray images taken at postoperative 7-year follow-up. **C**: Clinical photograph at postoperative 7-year follow-up

A 15-year-old boy with achondroplasia presented with Methicillin-Resistant Staphylococcus aureus (MRSA)-infected femoral nonunion. Before now, bilateral femoral lengthening was performed at age 13 for rhizomelic shortening of the femoral bones using a unilateral external fixator at his previous hospital (Fig. 1). After a 7 cm lengthening of the right femur, surgical site infection occurred around the limb lengthening, caused by MRSA from the skin, detected around the femoral pin and surgical site on postoperative day 127 at his previous hospital. The previous physicians considered MRSA colonization, not infection. They treated the patient with first-generation Cephem antibiotics; however, the condition did not improve. Subsequently, debridement was performed on postoperative day 145. After two pins were removed, four pins were kept in place, and two drains were placed at the sites of the removed two pins (Fig. 2). In addition, the hip spica casting was performed on the right femur. On postoperative day 271, they considered the MRSA eradicated, and they fixed a small-plate non-locking screw using an autologous iliac bone graft. The patient wore a brace for 13 months after the plate surgery. However, the nonunion did not heal. The patient was transferred to our hospital at age 15. In addition, the previous physician informed us that the MRSA had been eradicated at the time of transfer, though there was nonunion. Radiography and computed tomography showed hypertrophic nonunion of the femur. (Fig. 3). We considered residual deep infection at the limb lengthening. The femur was approached laterally, the plate was removed, and all the screws were loosened. The infected soft tissue around the nonunion site was thoroughly curetted and debrided. We further debrided the bone until punctate bleeding was seen, and placed cement beads with vancomycin [7]. Bone biopsies were obtained from several bone tissues at nonunion sites and a 5 mm diameter half pin was inserted into the femoral head to fix the femoral nonunion firmly. However, we noticed that the half-pin could not be used for fixation because the bone strength of the femoral head had reduced significantly. Consequently, the half-pin easily dislodged due to poor fixation. We anticipated a significant reduction in the bone strength of the right femur due to the patient’s two-year-long inactivity. To address this, we prepared a circular external fixator (220 mm ring), which can be firmly fixed even with reduced bone strength. Then, we meticulously and carefully placed multiple penetrating wires proximally and distally at the infected nonunion of the femoral lengthening site to avoid neurovascular injury, and no such injuries were observed (Fig. 4). Furthermore, during surgery, we applied a single ring to the proximal lower leg to increase the fixation strength and performed joint-spanning fixation across the knee joint (Fig. 4). The patient was overweight (weigh: 58.9 kg, height: 139.0 cm), and had a BMI of 30.5; however, he was allowed to walk carefully with partial weight bearing using a walker after surgery (Fig. 5). A knee joint-spanning external fixator was used, the tibial ring was removed two weeks after surgery, and range-of-motion exercises were initiated. Notably, bone tissue culture around the nonunion site revealed MRSA. We selected daptomycin based on antibiogram guiding. We administered daptomycin at an adult dose of 6 mg/kg/day for 28 days because the patient weighed 58.9 kg. The patient experienced no side effects due to the antibiotics. In addition, the tissue around the nonunion was thoroughly curetted and debrided again, and the cement beads with vancomycin 6 weeks after circular external fixation, as scheduled. There was no apparent tissue contamination around the nonunion site. Good intramedullary bleeding was observed at the nonunion site and MRSA was not detected in several tissue samples from the nonunion site. After 6 weeks, the final fixation was performed as scheduled. We replaced all rings and wires, thoroughly freshened the nonunion site, and placed a cancellous bone graft from the contralateral iliac bone (Fig. 6). The bone union was delayed; however, its fusion progressed gradually, and the ring was removed 9 months after the final surgery.　No recurrence of the infection was observed 7 years post-surgery. This patient had a slight limitation of flexion in his right knee; however he did not experience pain while walking, now a healthy working adult (Fig. 7).

## Discussion and conclusions

Bilateral lower extremity lengthening has been performed in patients with achondroplasia. However, there are no detailed treatment reports for deep infections occurring around the limb lengthening. The present case involved a 13-year-old boy who was short but originally lived normally. He was referred to our hospital after a 2-year treatment for a deep infection resulting from femoral lengthening surgery at another hospital. Infection is a complication in patients with achondroplasia, requiring careful precautions. Deep infections were the obstacles to femoral lengthening [5, 8–10], and successful infection eradication relies on thorough debridement of all infected and necrotic tissues [11, 12]. The highest cure rates are achieved with a two-stage debridement procedure, antibiotic beads, and planned secondary fixations [13]. Masrouha KZ et al. reported that the surgical procedure is an integral part of the treatment protocol, and emphasize the role of thorough debridement of any necrotic tissue before the application of beads [13]. Multiple small and round antibiotic beads provide a greater surface area with a higher percentage and rate of antibiotic release than a single antibiotic bead of the same volume. One clinical trial showed that approximately 5% of the antibiotic is released in the first 24 h, with amounts subsequently released gradually decreasing to a minimal concentration over several months [14]. However, Nelson et al. showed that the amount of antibiotic released from antibiotic beads during the first 10 weeks post-implantation is much higher than the antibiotic released after 14 weeks [15]. The previous physician failed to identify the bacteria in the infected nonunion site. Furthermore, prolonged antibiotic therapy aimed at reducing inflammation and improving clinical symptoms, without prior revision surgery, has not been effective in eradicating the infection. Moreover, it is possible that the infection could not be eradicated due to under-debridement. Appropriate debridement is crucial for successfully treating infected nonunion [7, 11]. The significant loss of bone strength from 2 years of non-weight-bearing complicated the treatment. However, we were unable to fix the proximal femur with half pins and managed to fix it with a circular external fixator. Placing the through-wire in the proximal femur made avoiding the neurovascular bundle challenging and required an advanced technique. Notably, using a large number of penetrating wires for fixing the Ilizarov rings was not recommended by the Ilizarov surgeons due to the high risk of neurovascular injury. It is better to avoid the use of penetrating wires in the femur because of this risk. However, the bone strength was significantly reduced, making it difficult to fix with several pins.

We achieved firm fixation using multiple penetrating wires with 130 kg tension on the proximal and distal rings. We began physical therapy involving partial weight bearing using a walker on the operated leg immediately after surgery. The patient was able to walk without any support 1 week later. The patient’s hospital stay lasted 28 days after treatment using daptomycin. After discharge, the patient was healthy and enjoyed school life with the circular external fixator. He continued to attend school and was hospitalized for surgery, after which he was allowed to walk without crutches. The frame was removed nine months postoperatively, and the patient recovered anatomically and functionally. Seven years postoperatively, there was no leg-length discrepancy or angular malalignment of the lower extremities as determined clinically and radiographically (Fig. 7). Several reports have described that internal fixation is better tolerated by patients with lower morbidity and better (although delayed) mobility; however, it is associated with complications such as delayed weight-bearing, implant failure, need for further surgery, and lower mobility in the knee and ankle [16]. In addition, he had a slight limitation in the range of knee joint motion compared to the opposite side; on the contrary, he had no problems with activities of daily living.

There is no consensus regarding antimicrobial therapy for MRSA-infected nonunions. Few reports have been published, especially in children, and evidence is lacking. We treated an MRSA-infected nonunion of the femoral lengthening site without side effects using debridement, antibiotic beads, external fixations, and daptomycin administration.

From this case report, we showed that radical debridement, irrigation, antibiotic cement beads, and autologous iliac bone graft with circular external fixator application can successfully treat MRSA-induced nonunion of the femoral lengthening site in achondroplasia. The reported procedure is a reliable and efficient method for treating this MRSA-infected nonunion in achondroplasia; however, technically demanding. It also results in adequate healing time, immediate ambulation, and no complications. Based on the final clinical and radiographic outcomes, this technique proved acceptable for managing MRSA-infected nonunion of the femoral lengthening site in patients with achondroplasia.

## Data Availability

No datasets were generated or analysed during the current study.

## Abbreviations

MRSA: Methicillin-Resistant Staphylococcus aureus
BMI: Body mass index
